# An Online Data-Driven Fault Diagnosis Method for Air Handling Units by Rule and Convolutional Neural Networks

**DOI:** 10.3390/s21134358

**Published:** 2021-06-25

**Authors:** Huanyue Liao, Wenjian Cai, Fanyong Cheng, Swapnil Dubey, Pudupadi Balachander Rajesh

**Affiliations:** 1 SJ-NTU Corporate Lab, Nanyang Technological University, Singapore 637335, Singapore; huanyue.liao@ntu.edu.sg (H.L.); Fanyong.cheng@nut.edu.sg (F.C.); 2 School of Electrical and Electronic Engineering, Nanyang Technological University, Singapore 639798, Singapore; 3 Energy Research Institute @ NTU, Singapore 637141, Singapore; sdubey@ntu.edu.sg; 4 Surbana Jurong Consultants Pte Ltd., Singapore 150168, Singapore; rajesh.balachander@surbanajurong.com

**Keywords:** convolutional neural network, HVAC system air handling unit, fault diagnosis

## Abstract

The stable operation of air handling units (AHU) is critical to ensure high efficiency and to extend the lifetime of the heating, ventilation, and air conditioning (HVAC) systems of buildings. In this paper, an online data-driven diagnosis method for AHU in an HVAC system is proposed and elaborated. The rule-based method can roughly detect the sensor condition by setting threshold values according to prior experience. Then, an efficient feature selection method using 1D convolutional neural networks (CNNs) is proposed for fault diagnosis of AHU in HVAC systems according to the system’s historical data obtained from the building management system. The new framework combines the rule-based method and CNNs-based method (RACNN) for sensor fault and complicated fault. The fault type of AHU can be accurately identified via the offline test results with an accuracy of 99.15% and fast online detection within 2 min. In the lab, the proposed RACNN method was validated on a real AHU system. The experimental results show that the proposed RACNN improves the performance of fault diagnosis.

## 1. Introduction

Heating, ventilation, and air conditioning (HVAC) systems are the major energy consumers in the building, which contribute to over 40% of the energy consumption [1,2,3,4]. The reliability and availability of the HVAC system needs to be further improved in order to increase energy efficiency with proper operation and maintenance [5,6,7]. The large-scale HVAC systems have complex structures and usually work under unpredictable weather conditions and different indoor user settings [2]. Therefore, the actual operating state of the HVAC system is rather complex, and the features of the state are variable with strong nonlinearity and coupling characteristics [8]. Additionally, the HVAC systems include large sums of equipment such as dampers, sensors, controlled actuators, and so on. If maintenance is not implemented in a timely manner if a minor fault happens, this will reduce the efficiency, accelerate the equipment deterioration, and even damage other components [9]. Studies have shown that the highest failure of HVAC systems can lead to an increase in energy consumption of 30% [10]. Hence, it is essential to apply the fault diagnosis or the HVAC system to improve its reliability and performance. The air handling unit (AHU) is the main functioning component of the entire HVAC system, which is responsible for controlling the temperature, humidity, flow rate, and so on. The reliability of AHU is critical for the operation and maintenance of the HVAC system [11]. Accurate and fast fault detection and diagnosis are highly desirable for AHU in HVAC systems.

Fault detection and diagnosis have always been critical problems in the efficient and reliable operation of HVAC and energy optimization. In the past 20 years, research on fault diagnosis of HVAC systems has made significant progress [12]. Many scholars have adopted different model methods to realize the fault diagnosis of HVAC systems. In the review of the difference in the existing literature, the HVAC system fault diagnosis methods are considered as three basic models: quantitative model-based, qualitative model-based, and data-based process methods [13]. The division is based on the different modeling processes, divided into prior knowledge or data-driven information. The quantitative model-based and the qualitative model-based method rely on accurate system modeling and require experts to describe the detailed dynamic model of the entire HVAC system. However, the simulation model might have difficulty in considering all the details in practice. The data-driven methods can be stored and collected easily through the appropriate sensors, and the model does not require physical principles and knowledge. This feature makes the data-driven methods more practical when the models are not applicable, especially for large HVAC systems [14]. Furthermore, accurate online fault detection and diagnosis methods are critical for dealing with temporal data from the HVAC system [15].

Many model-based fault diagnosis methods have been proposed for HVAC systems [16]; however, this method is not applicable for the online fault diagnosis process for the slow response and limitation on the transient-state conditions [15]. The data-driven approaches have received more attention for online fault diagnosis applications, especially for the feature selection reduced the complex calculations [17]. Online fault diagnosis methods still need further development for higher accuracy and faster responses. 

Different data-driven approaches have been developed for fault diagnosis of AHU in HVAC systems, such as neural networks (NN) [11], support vector machine (SVM) and random forest (RF) [16], the principal components analysis (PCA)-based model [18], and the decision tree based supervised learning [19]. In addition, there are hybrid methods that combine expert knowledge and data-driven methods such as [20,21] based on the Bayesian Networks with the rule-based approach. For the NN-based model, the hyperparameters are relatively more than other data-driven methods, and the performance for the diagnosis is limited. For the SVM-based model [22], the kernel function is hard to match multiple signals with different scales. For the PCA-based method, it is hard to generate and present the abstract features of the multiple signals with different scales through linear mapping. The heavy training and computation for the data-driven methods need to further attention, and the accuracy of the diagnosis requires further improvement. Additionally, the above-mentioned methods are only tested with the historical data; the performance of the online fault diagnosis of AHU is not verified.

Among data-driven methods, convolutional neural networks (CNNs) is a popular supervised-learning algorism, which is commonly used for computer vision and image processing applications due to their computation efficiency, high-performance accuracy, and feature extraction, and classification fused characteristics when dealing with high-dimensional data [23]. Many papers proposed the CNN-based fault diagnosis methods related to the bearing defects in rotary machinery [24]. In [25], the modified cost function with the additional sparsity cost is established in the proposed CNN-based fault diagnosis methods for better performance with fewer input data. In [26], the transfer CNN-based fault diagnosis method is proposed for adaptability to new operating conditions. In [27], a proposed CNN-based fault diagnosis method is proposed for compound fault diagnosis. 

The merit of CNN is better feature extraction for complex systems. One-dimensional convolutional neural network (1D CNN) is applied for bearing fault detection in [28], motor fault detection in [29], and fault diagnosis for modular multilevel converter (MMC) circuits in [30]. Additionally, the 1D data can be applied with CNN for condition monitoring and fault diagnosis [29,30,31]. 1D CNNs can capture the features from the fast signals in short fixed-length segments. There are some methods proposed for transferring the raw data into a 2-dimensional configuration to fit with the 2D CNNs algorism [32,33]. 

In this paper, a novel online fault diagnosis method is proposed for AHU systems, called the rule and convolutional neural networks (RACNNs) method. This method aims to address the limitations in the diagnosis accuracy of the previous works by adopting the recently evolving topology of CNN. Therefore, RACNN is proposed to combine the advantage of the fast speed of the rule-based method for sensor data and the advantage of the high accuracy of CNN for system fault detection.

The proposed online automatic fault diagnosis and detection method for AHU in the HVAC system receives the following contributions in this paper:

(1). A novel online fault diagnosis approach for the AHU system using 1D CNN in combination with the rule-based model is proposed. The model is established through the historical data obtained from the building management systems. Prior knowledge of the AHU system model and the reason for the fault is not required, which is beneficial for non-specialists;

(2). The rule-based model is introduced to constitute a primary model for the sensors’ operation state, and the 1D CNN model constitutes a senior model to detect the complex fault. The detection performance and the speed are enhanced through the proposed method;

(3). The proposed RACNN method was validated on a real AHU system. The common faults for the AHU system are all considered. The experimental results demonstrate that the proposed RACNN significantly improves the performance of fault diagnosis.

## 2. Model Description

The proposed online automated fault diagnosis method is based on the data collected in the real HVAC system with AHU. A detailed system description is offered below.

### 2.1. Air Handling Units System

**Figure 1:**
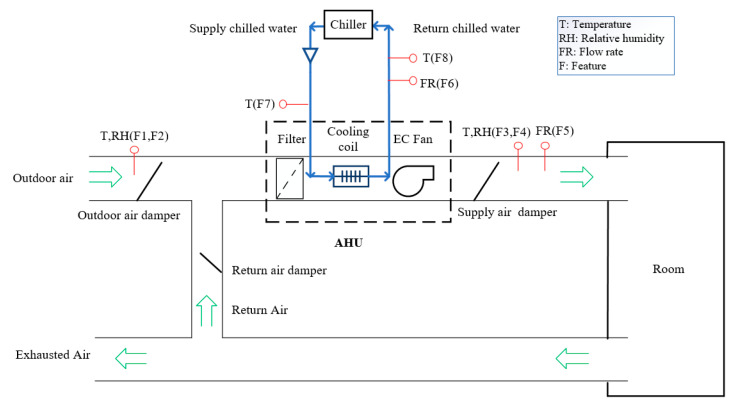
The schematic diagram of the experimental air handling units (AHU) system.

The schematic diagram of the experimental AHU system is presented in Figure 1. 

**Figure 2:**
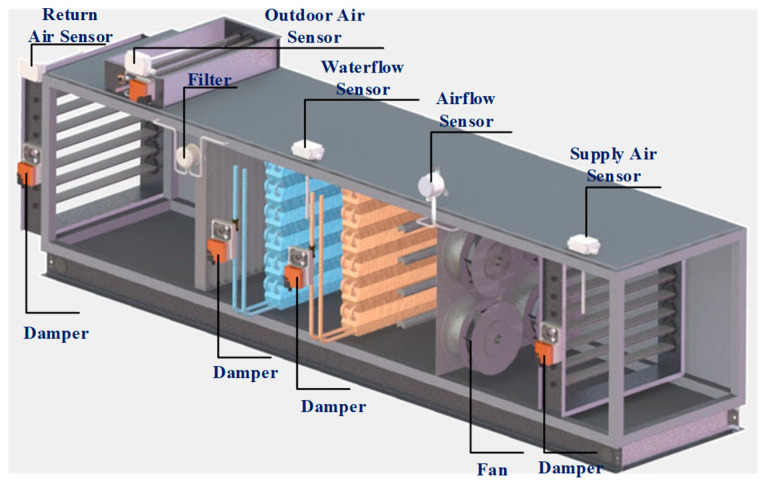
The structure diagram of the air handling unit (AHU) in the laboratory.

The AHU system is an important piece of equipment in HVACs, and has a major impact on the ventilation performance of the HVAC system. The AHU is the place where the water-cooling system and the air system exchange heat. A structural diagram of the AHU in the laboratory is shown in Figure 2. The AHU mainly consists of the supply air valve, return air valve, outdoor air valve, filter, cooling coil and its valve, fans, and the control system. The heating mode is not considered in this case for lack of the heating coil. The outdoor air enters the building and mixes with the returning air through the AHU. The outdoor air valve and the return air valve are controlled for the proper percentage of the mixed air volume to ensure indoor air quality. Then, the mixed air is purified through the filter and chilled through the cooling coil. The cooling coil valve is controlled to achieve the setpoint of the supply air temperature. The cooling coil water temperature is maintained at 10 °C. The air volume is controlled by the speed and the installation angle of the fan and the valves. Finally, the purified and chilled air is supplied to the variable air volume (VAV) system or directly to the building.

The main signals of the AHU system are presented in Table 1. The main sensor measurements include the return air temperature Rt (°C), the return air humidity Rh (%RH), the chilled water tank temperature Cst (°C), the supply air temperature St (°C), the supply air humidity Sh (%RH), the airflow rate (m^3^/h), the outdoor air temperature Ot (°C), and the outdoor air humidity Oh (%RH). The control signal for the supply air temperature setpoint is 22 °C. The amplitudes of the sensors are also presented in Table 1.

### 2.2. Fault Analysis of AHU System

The AHU is the core component of the whole HVAC system, and it is also the most fragile part. The failure of the AHU system has attracted much attention for improving the efficiency and the performance of the HVAC system. In [34], the fault analysis for the AHU system was investigated. The air valve failure fault, the water failure valve fault, and the fan failure fault are the most common faults in the AHU system. The common faults of the AHU system are summarized in Table 2. The proposed online fault diagnosis and detection methods will consider all the faults mentioned in Table 2.

Excessive air leakage will decrease the outdoor air to the system. The outdoor air damper stuck will then lose control of the airflow rate, therefore decreasing the efficiency of the AHU system. The effects of the above-mentioned two faults are very similar, therefore an accurate diagnosis will be difficult, especially for the people who are not familiar with the overall system. The sensor faults also need to be considered for improving the accuracy of the fault diagnosis for the AHU system.

### 2.3. HVAC System

**Figure 3:**
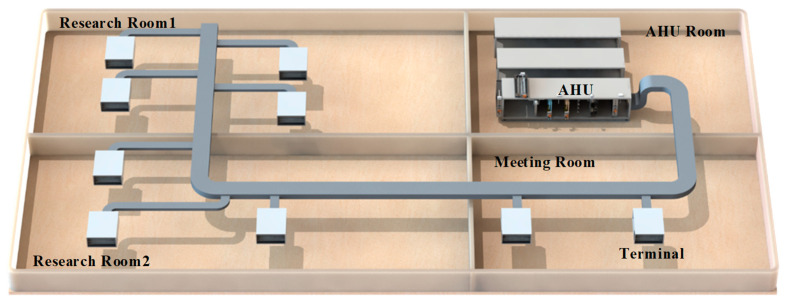
Structure of the experimental platform of the HVAC.

A test HVAC system with an AHU system was fabricated in the laboratory. The HVAC system provides cooling air for a floor of an office located in Nanyang Technological University, as shown in Figure 3. The operating hours are from 8 a.m. to 6 p.m. on weekdays. The floor of the office is composed of four zones and the total volume is 240 m^3^. The AHU room is where the test AHU system is located, while the other rooms are the office rooms. The return air from the three offices is collected and sent to the AHU through the return air ducts. The dampers for the exhaust air, the outside air, and the return air are controlled simultaneously to maintain the indoor air quality.

The temperature of the cold air is around 12 °C from the AHU supplied to the offices. The flow rate is controlled through intelligent terminals with smart dampers. The temperature of the cooling water is around 10 °C from the chiller and it connects to the cooling coil through the chilled water tank, which is regulated by the water valve.

## 3. Fault Diagnosis Methodology

**Figure 4:**
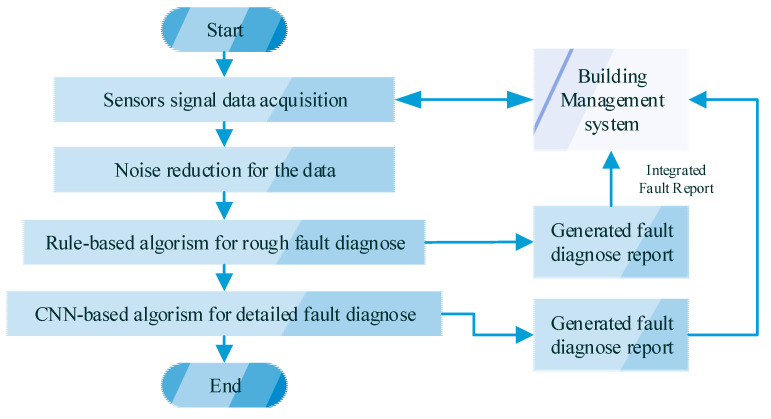
Overall fault diagnostic flowchart for the AHU system.

The overall fault diagnosis method for the AHU system is shown in Figure 4. The proposed online fault diagnosis and detection method combine the rule-based method and one-dimensional convolutional neural network-based method.

Firstly, historical data are collected through the sensors and integrated by the information management system (BMS). Then, the noise of the sensor data needs to be filtered to purify the necessary information. Finally, the rule-based and convolutional neural network method (RACNN) is applied for fault diagnosis and detection. The rule-based method is applied for the initial fault diagnosis, especially for the sensor state diagnosis. The thresholds of the rules are all defined by the normal historical data and the algorithm; therefore, prior knowledge is not required in this part. In addition to this, the rule-based methods can enhance the accuracy of the final diagnosis when the sensors are stuck or drift from the normal operation. The 1DCNN can detect and diagnose the fault with high accuracy, which is of especial benefit for distinguishing similar fault cases, such as excessive air leakage and outdoor air damper stuck.

An analytical model of fault diagnosis and detection follows the modular design for different types of AHU systems and components. In this paper, the RACNNs are applied for the fault diagnosis of the AHU system. Therefore, the reliability of the AHU and the overall HVAC system can be further improved by this fast and accurate fault diagnosis method.

### 3.1. Signal Noise Reduction

The pre-processing of noise separation for the sensor data is necessary to improve the performance of the fault diagnosis and detection. The one-dimensional non-local means filtering algorithm is applied for signal filtering in this fault diagnosis and detection method. All the collected data will be applied with this filter to separate the noise and purified data. The non-local means consider all the data of the signals and add the white noise during the denoising process. The data filtered by the non-local means algorithm experience less loss than the local mean algorithm.

The signal with the white Gaussian noise is presented as:

(1) y(i)=x(i)+n(i)

where y(i) represents the collected noise signal, x(i) represents the theoretical original noise-free signal, and n(i) represents additive white Gaussian noise.

The non-local mean filtering algorithm $\tilde{x}$ is used to represent the estimate of the original signal *x*. This estimation is obtained by the weighted average of all similar structural data blocks in the search window. Therefore, the non-local mean algorithm can theoretically be expressed as follows:

(2) $$\tilde{x}(s)=\frac{1}{Z(t)}\sum_{t\in M(s)}\omega (s,t)y(t)$$

where ω(*s*,*t*) represents the similarity degree of a similar structural block centered on t, with the target structural block centered on *s* in the search area of the signal, *M*(*s*).

(3) $$\begin{array}{l} \omega (s,t)=\exp\left(-\frac{\sum_{\delta \in A}(y(s+\delta )-y(t+\delta ){)}^{2}}{2L_{\Delta }\lambda^{2}}\right)\\ \;=\exp\left(-\frac{d^{2}(s,t)}{2L_{\Delta }\lambda^{2}}\right) \end{array}$$

represents the calculation method of the weight (similarity degree) in the search area, s, centered on the target block. *Z*(*t*) represents the sum of the similarity degrees of all similar structural blocks, which is a standard constant. Where *λ* represents the filter parameter, Δ represents the target structure block centered on *s*, and *L* presents the similar structural block at the center *t*.

The non-local mean algorithm can effectively remove the interference of Gaussian noise, and its denoising effect is better than that of most conventional algorithms, such as wavelet denoising and singular value denoising.

### 3.2. Proposed Rule-Based Diagnosis Technique

The states of the sensors are important for the data collection; therefore, their state will also influence the fault diagnosis for other components of the AHU system. The rule-based algorithm is proposed for rough identification for the sensor’s operation states. The diagnose thresholds are determined according to the historical data and the statistical analysis method. For example, if only the airflow sensor is stuck or broken, the algorithm may decrease its accuracy in the diagnosis of the performance of the fan.

The reference value deduced from the normal operation historical data is called the fault detection threshold. The selection of the fault detection threshold is related to the sensitivity of the false alarm rate. If the fault detection threshold is larger than the normal operation range, the fault diagnosis system may be insensitive to small faults, which may cause the failure to report false alarms; if the fault detection threshold is too small, the fault diagnosis alarm system may trigger more false alarms.

The residual between the model-predicted value and the actual measured value of the system is calculated, and the residual is then compared with the threshold deduced from the normal operation. If the residual does not exceed the threshold, then the sensors are well-operated, otherwise, the system is faulty or abnormal. An online adaptive estimation method of the fault detection threshold is proposed to determine the rule-based diagnosis for sensor states. Modeling errors and measurement errors are updated with changes in the operating conditions of AHU, and the fault detection threshold can be calculated using statistical methods under a certain level of confidence. The threshold is deduced as:

(4) $$Thr_{0,i}=U\left({\tilde{r}}_{i}\right)=t_{\alpha /2,n-p}{\tilde{\sigma }}_{{\tilde{r}}_{i}-r_{i}}$$

where $Thr_{0,i}$ is the fault detection threshold for the output variable of the model *i*; $r_{i}$ is the output variable of the model *i*; ${\tilde{r}}_{i}$ is the residual estimation of the output variable of the model *i*; $t_{\alpha /2,n-p}$ is the *t* distribution value under the degree of freedom (*n* − *p*) at the confidence level (1 − α); *n* is the modeling data quantity; *p* is the number of the input variables of the model.

(5) $${\tilde{\sigma }}_{{\tilde{r}}_{i}-r_{j}}=\underset{Modeling\;error\;estimation}{\underbrace{{\tilde{\sigma }}_{Y_{j}}^{2}\left[1+x_{0}^{T}{\left(X_{reg}^{T}X_{reg}\right)}^{-1}x_{0}\right]}}+\underset{Measurement\;error\;estimation}{\underbrace{\sum_{j}{\left[\left(\frac{\partial g_{i}}{\partial z_{j}}\right)\sigma_{z_{j}}\right]}^{2}}}$$

where ${\tilde{\sigma }}_{Y_{j}}^{2}$ is the sum of the variance of the residuals between the predicted value and the measured value of the model *i*; $x_{0}$ is the row vector of the sensors operating data (a set of model input data); $X_{reg}$ is the matrix of the model established by the normal operation data.

### 3.3. Convolutional Neural Networks Fault Diagnosis Method

The neural network can fully fit the nonlinear relationships by organizing and updating the neurons automatically; therefore, this method has been widely used in many applications. With the development of deep learning, many advancing structures of neural networks have been proposed in previous research. CNN is one of the deep neural networks that have attracted great attention as regards pattern recognition. CNN is a feedforward neural network, and it can extract the local features even with deformations. CNN has always been used for two-dimensional modeling in speech recognition or image identifications. The convolution processes are developed in two dimensions with completely different physical meanings, which cannot adapt well to the one-dimensional characteristics of the signals used for the fault diagnosis. The 1D CNN model used for the fine fault diagnosis and detection of the AHU system has been proposed in this paper.

#### 3.3.1. One-Dimensional Convolutional Neural Networks

CNN is a feedforward neural network, and the neurons are arranged hierarchically. The general structure of CNN consists of the input layer, hidden layer, and output layer. All neurons are passed from the previous network to the next network without feedback. The hidden layer of CNN is a multi-layer network, and it generally includes a multi-convolutional layer and a pooling layer. The deep structure of CNN can extract the local feature with high robustness and accuracy.

**Figure 5:**
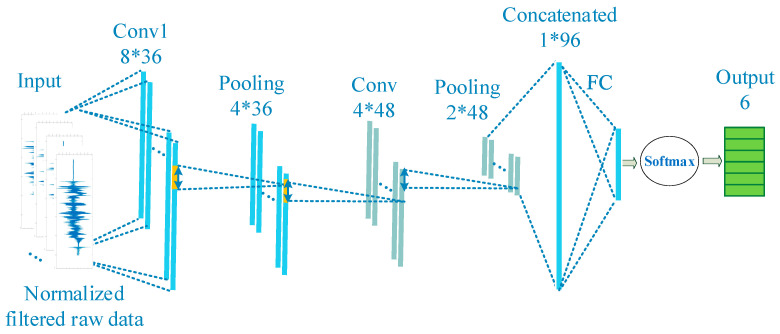
One-dimensional convolutional neural network framework.

To retain the one-dimensional features of the original signals, the 1D CNN algorithm is proposed for fine fault diagnosis and detection in the AHU. The principle is to sequentially extract relevant feature vectors from the original time series-related signals. As shown in Figure 5, its input is a one-dimensional feature vector, so its internal convolution kernel and feature map are all one-dimensional. The signals are arranged and normalized into the standard forms and formulated as the input layer.

Then, through the convolutional layer, the features of the signals can be extracted with the convolutional kernel [35]. The convolutional process traverses the original signals with the designed kernel and step sizes. In each step, the convolution calculation with the convolutional kernel and the data in the original signals within the batch is generated. The convolutional process is presented as:

(6) $$X_{i,k}=\sum_{b=1}^{n-1}W_{b,k}S_{b+i}^{T}+b_{k}$$

where $X_{i,k}$ presents the input of the *k*-th convolutional kernel in the *i*-th group, *n* represents the batch of the convolutional kernel, $S_{b+i}^{T}$ represents the input feature vector of the *i*-th group, $W_{b,k}$ represents the weight of the *k*-th convolutional kernel, and $b_{k}$ represents the network bias.

The convolutional result presents the locally extracted features and the important information. The number of kernels is a hyperparameter that needs to be tuned.

The 1-D forward propagation (FP) can be expressed as:

(7) $$x_{k}^{l}=b_{k}^{l}+\sum_{i=1}^{N_{I-1}}\mathrm{conv}1D\left(w_{\mathrm{ik}}^{l-1},s_{i}^{l-1}\right)$$

where $x_{k}^{l}$ is the input, $b_{k}^{l}$ is a scalar bias of the *k*-th neuron at layer *l*, and $s_{i}^{l-1}$ is the output of the *i*-th neuron at layer *l* − 1. $w_{ik}^{l-1}\;$ is the kernel from the *i*-th neuron at layer *l* − 1 to the *k*-th neuron at layer *l*. The number of neurons *N* in the fully connected (FC) layer is a hyperparameter to be set in addition to the type of activation function.

The rectified linear unit (ReLU) activation function is commonly used with CNNs. It returns 0 if the input is negative. The linear separability of the features occurs through the activation functions. The expression of ReLU is

(8) $$a_{i}^{l+1}=f\left(x_{i}^{l+1}\right)=\max\left\{0,x_{i}^{l+1}\right\}$$

where $a_{i}^{l+1}$ presents the activation value of $x_{i}^{l+1}$, and $x_{i}^{l+1}$ is the output value after the convolution process.

The pooling layer is designed for preventing overfitting for the algorithm and reduces the computational complexity. The pooling process enhances the robustness of the CNN. Max-pooling is applied in this method.

Through the FC layer, the feature extracted through the convolutional layer and the pooling layer can be concatenated and mapped to the sample label space. Finally, the classification of the operation condition is predicted through the output layer. The softmax classifier is applied as a general output layer. The probability of each fault case can be deduced through the logistic regression as:

(9) $$P_{j}=\mathrm{softmax}\left(z_{j}^{}\right)=\frac{e^{z_{j}^{T}}}{\sum_{k=1}^{A}e^{{}^{z_{j}^{T}(k)}}}$$

where $P_{j}$ is the probability of the output vector of the *j*-th neuron at the output, $z_{j}^{}$ is the weight parameter, and A is the total number of the categories.

The framework of complex fault is detected by the 1D CNN-based fault diagnosis method, as illustrated in Figure 5. The hidden layer contains two convolutional layers and two pooling layers. The input batch size is eight data points of the signal sample. Then, there are 36 kernels for the first convolutional layers and 48 kernels for the second convolutional layer. The kernel size is two for all the convolutional layers. The length of the pooling layer is two and the step is two. The feature size is 64 through all the layers. In this case, there are four types of fault cases simulated in the lab. The output layer size is five, including four fault conditions and normal operating conditions.

The hyperparameters in 1D CNN mainly include the learning rate, the number of epochs, the batch size, the number of the hidden layers, and the units and weight initialization. The hyperparameters need to be designed based on the structures of the data and minimizing the cost function.

The cost function is optimized through the cross-entropy function:

(10) $$E=\sum_{i=1}^{N}\sum_{j=1}^{C}t_{ij}y_{ij}$$

where *t_ij_* is the *j*-th element of the target one-hot vector *t_i_*, and *y_ij_* is the *j*-th element of the corresponding output vector *y_i_*.

#### 3.3.2. Proposed Online Fault Diagnosis Framework

**Figure 6:**
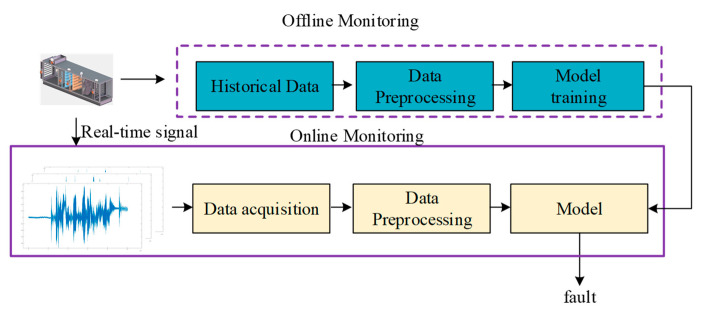
The Online AHU system fault diagnosis method framework.

The framework of the online RACNN diagnosis method is proposed in Figure 6. The framework includes two stages: an offline stage and an online stage. The offline stage provides the trained model based on the historical operation data of the AHU system through the designed 1D CNN algorithm. The online stage can detect and diagnose the operating conditions based on the real-time measurements of the system and the model from the offline stage.

The random search for the hyperparameter is applied to effectively design the best-performing network. The Adam optimization algorithm with piecewise learning decay is applied to optimize the cost function in the 1D CNN. The learning rate is initialized to 0.001, and the epoch is set to 30.

## 4. Case Studies

### 4.1. Experiments

**Figure 7:**
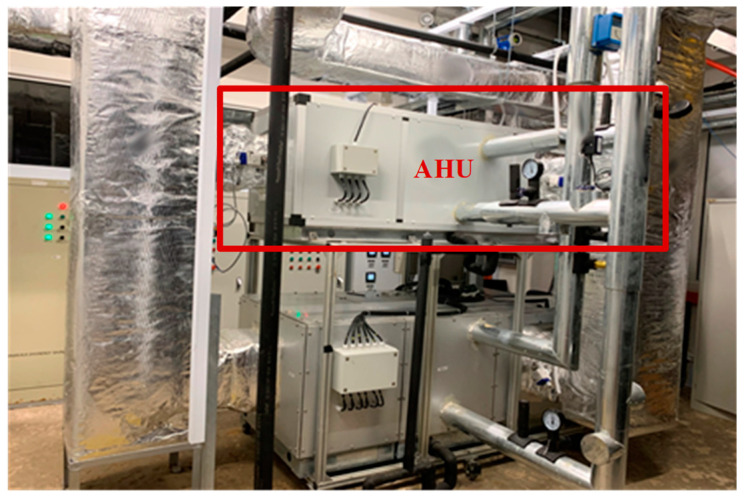
The experimental platform for the test of the AHU in the heating, ventilation, and air conditioning (HVAC) system.

The AHU system is executed under one normal condition and four fault conditions, as listed in Table 3. The experiment platform is shown in Figure 7. The fault cases are manually simulated in the lab. All the faults are simulated under three conditions. The cooling coil valve is manually switched between three different angles to mimic the cooling coil valve stuck fault. The speed of the fan is manually slowed down to three different speeds. The leakage area of the duct is designed for three different scales for the unit air leakage fault.

### 4.2. Data Collection

The total dataset is 6048 samples, which includes 1324 samples for the normal operating condition. The summary of the sample size for each type of fault is shown in Table 1. For real applications, it may be hard to find the fault samples, and the normal states are more frequent. The classes will be imbalanced. The main signals are listed in Table 1. It can be found that it is difficult to distinguish different faults using only each sensor signal separately; therefore, it is necessary to apply the advanced fault diagnosis method of RACNNs for the AHU system. AHU data were collected in 35 s time intervals, which is also the sampling frequency. The operating hours were from 8 a.m. to 6 p.m. on weekdays. The total experiments lasted 3 weeks.

It should be noted that the fault diagnosis for AHU can also be considered as a multiclass classification problem. In this case, there are five classes for all the samples. The algorithm is applied by a laptop with 64 GB RAM and an Intel(R) Core (TM) i7-7700HQ CPU processor at 2.8 GHz speed.

### 4.3. Performance Metrics

To compare the performance of the classification, the *F*_1_ score is adopted as the metric for different methods, which can be defined as:

(11) $$F_{1}=\frac{2\mathrm{TP}}{2\mathrm{TP}+\mathrm{FP}+\mathrm{FN}}$$

where TP (True Positive) is the number of positive samples with a correct classification result, FP (False Positive) is the number of positive samples with a faulty classification result, TN (True Negative) is the number of negative samples with a correct classification result, and FN (False Negative) is the number of negative samples with a faulty classification result. In the following studies, the *F*_1_ score for all the faults will be calculated with the proposed approach, and the results will be compared.

### 4.4. Validation and Cooperation

The proposed RACNN fault diagnosis methods are compared with other data-driven fault diagnosis methods in this section. Here, the proposed RACNN method is validated and compared with the NN-based method [11], and the SVM-based method [16]. The total data are split into two groups, with 40% for training and validation, and 60% for testing. The preprocessing of the signals, including normalization and denoising, is applied for the NN-based method, the SVM-based method, and the proposed RACNN-based method.

**Figure 8:**
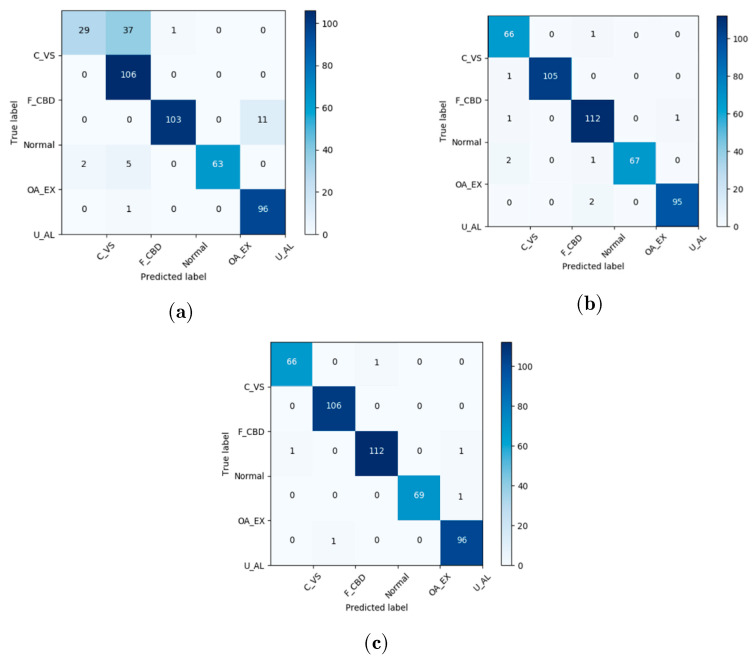
The confusion matrix of test samples: (**a**) neural network (NN)-based method, (**b**) support vector machine (SVM)-based method, and the (**c**) proposed rule and convolutional neural networks (RACNN)-based method.

The confusion matrix used to test the classification results of the abovementioned three methods is shown in Figure 8. The correctly classified samples and misclassified samples for each condition are presented through the matrix. The horizontal axis and vertical axis represent predicted labels and true labels, respectively. It is easily seen that only six fault samples are misclassified by the proposed RACNN method, 48 are misclassified by the NN-based method, and nine are misclassified by the SVM-based method. The accuracy of fault diagnosis through the proposed RACNN method reaches 99%. This implies that the proposed method can extract more discriminant features for different faults compared with other methods. The online diagnosis is also tested, and the detection can be made within 2 min, which is rather fast considering the sampling time is 35 s.

The comparison is also made with the NN-based and SVM-based fault diagnosis methods. The RACNN method achieves better performance than these methods. The accuracy for the proposed RACNN method is 98.6%, as mentioned before. The accuracy of the NN-based method and the SVM-based method is 89% and 98%, respectively. For the average sensitivity (recall), the RACNN method reaches 99%. The recall of both the NN-based methods and the SVM-based method is around 97%. There is a 2% improvement for the proposed method compared to the other two data-driven methods, which means a lower missed alarms rate. The reliability of the proposed RACNN is validated for the AHU in the HVAC system.

The comparison results among the proposed RACNNs and the NN-based and SVM-based methods are displayed in Table 4, in the form of the *F*_1_ score. The RACNNs method achieves the best overall performance: over 98% for all kinds of faults.

## 5. Conclusions

This paper proposes a new rule-based method and CNNs-based method (RACNN) framework for online automatic fault diagnosis of AHU under four fault conditions. The rule-based method can automatically and effectively detect the sensor condition by setting threshold values according to historical data and statistical methods. Accordingly, a 1D CNN-based framework of fault diagnosis is proposed, which does not rely on handcrafted features. It can perform automatic feature extraction and fault classification simultaneously, without depending on complex signal processing algorithms and prior knowledge. Experimental results in the real laboratory demonstrate that the proposed RACNN improves the performance of fault diagnosis. The proposed framework can maintain reliable operation and reduce the consumption of energy within the AHU system. It offers a new general-purpose framework for fault diagnosis, and it can be easily extended for different applications such as machines and industrial systems.

## Figures and Tables

**Table 1:** The main signals of the AHU system.

Index.	Variable	Description	Range
1	Rt	The return air temperature	22–25 °C
2	Rh	The return air humidity	60–82 %RH
3	Cst	The chilled water tank temperature	10–11.5 °C
4	St	The supply air temperature	12–12.5 °C
5	Sh	The supply air humidity	80–88 %RH
6	Sf	Airflow rate	700–760 m^3^/h
7	Ot	The outdoor air temperature	26–28 °C
8	Oh	The outdoor air humidity	78–82 %RH

**Table 2:** The main faults of the AHU system.

Label	Fault Description	Fault Devices
1	Cooling coil valve stuck	Cooling coil valve
2	Fan broke down	Fan
3	Outdoor air damper stuck	Damper or control
4	Air leakage	Duct
5	The return air temperature sensor failure	The return air temperature sensor
6	The return air humidity sensor failure	The return air humidity sensor
7	The chilled water tank temperature sensor failure	The chilled water tank temperature sensor
8	The supply air temperature sensor failure	The supply air temperature sensor
9	The supply air humidity sensor failure	The supply air humidity sensor
10	Airflow rate sensor failure	Airflow rate sensor

**Table 3:** Description of AHU conditions.

Label	Fault Type	Fault Description	Sample Size
1	C_VS	Cooling coil valve stuck	852
2	F_CBD	Fan circuits broke down	1324
3	Normal	Normal condition	1324
4	OA_EX	Outdoor air excessive	968
5	U_AL	Unit air leakage	1580

**Table 4:** Comparison results of the *F*_1_ metric.

Method\Fault	C_VS	F_CBD	Normal	OA_EX	U_AL	Min. *F*_1_
NN	0.592	0.831	0.945	0.947	0.941	0.592
SVM	0.963	0.995	0.9738	0.978	0.995	0.963
RACNNs	0.987	0.995	0.987	0.993	0.985	0.985

## Data Availability

The data presented in this study are available on request from the corresponding author. The data are not publicly available due to confidential requirement of the funding project.
